# TLR5 Signaling Enhances the Proliferation of Human Allogeneic CD40-Activated B Cell Induced CD4^hi^CD25^+^ Regulatory T Cells

**DOI:** 10.1371/journal.pone.0067969

**Published:** 2013-07-03

**Authors:** Ping-Lung Chan, Jian Zheng, Yinping Liu, Kwok-Tai Lam, Zheng Xiang, Huawei Mao, Yuan Liu, Gang Qin, Yu-Lung Lau, Wenwei Tu

**Affiliations:** Department of Paediatrics and Adolescent Medicine, Li Ka Shing Faculty of Medicine, University of Hong Kong. Hong Kong SAR, China; The Chinese University of Hong Kong, Hong Kong

## Abstract

Although diverse functions of different toll-like receptors (TLR) on human natural regulatory T cells have been demonstrated recently, the role of TLR-related signals on human induced regulatory T cells remain elusive. Previously our group developed an *ex vivo* high-efficient system in generating human alloantigen-specific CD4^hi^CD25^+^ regulatory T cells from naïve CD4^+^CD25^−^ T cells using allogeneic CD40-activated B cells as stimulators. In this study, we investigated the role of TLR5-related signals on the generation and function of these novel CD4^hi^CD25^+^ regulatory T cells. It was found that induced CD4^hi^CD25^+^ regulatory T cells expressed an up-regulated level of TLR5 compared to their precursors. The blockade of TLR5 using anti-TLR5 antibodies during the co-culture decreased CD4^hi^CD25^+^ regulatory T cells proliferation by induction of S phase arrest. The S phase arrest was associated with reduced ERK1/2 phosphorylation. However, TLR5 blockade did not decrease the CTLA-4, GITR and FOXP3 expressions, and the suppressive function of CD4^hi^CD25^+^ regulatory T cells. In conclusion, we discovered a novel function of TLR5-related signaling in enhancing the proliferation of CD4^hi^CD25^+^ regulatory T cells by promoting S phase progress but not involved in the suppressive function of human CD40-activated B cell-induced CD4^hi^CD25^+^ regulatory T cells, suggesting a novel role of TLR5-related signals in the generation of induced regulatory T cells.

## Introduction

Natural regulatory T cells (nTregs) and induced regulatory T cells (iTregs) are important to the self-tolerance of the human body and the tolerance to transplanted organs or tissues [1], [2]. Impairments in the development or functions of these cells can cause autoimmune diseases such as immunodysregulation polyendocrinopathy enteropathy X-linked syndrome [3], and systemic lupus erythematosus [4], which is either fatal or severely reduces the quality of life of patients, and graft rejection in transplantation. Although many efficient strategies have been developed to treat autoimmune diseases and graft rejection, their severe side effects lead to an urgent need for novel therapeutic strategies, such as adoptive transfer of antigen-specific regulatory T cells [5]. As a result, investigation in the biology of regulatory T cells is crucial for understanding these diseases and the development of novel therapeutic strategies for treating and managing autoimmune diseases and graft rejections.

It is known that activation and function of regulatory T cells require signals from both T cell receptor (TCR) [6] and CD28 [7], [8]. However, as increasing number of co-stimulatory molecules, such as OX-40 and PD-1, were discovered to be implicated in the activation and function of regulatory T cells [9], [10], it is speculated that co-stimulatory molecules may also play diverse and crucial roles in the activation and function of these cells [11]. Reports about the non-absolute requirement of TCR signal in T cell function further support this speculation [12], [13]. As a result, investigation in the role of co-stimulatory molecules in regulatory T cells is warranted. Although toll-like receptors (TLR) are thought to mainly participate in the antigen recognition and activation of innate immune cells [14], they are also crucial co-stimulatory molecules involved in the function of T cells. *In vitro* data suggested that TLR2, 4, 5, 7, and 8 could promote the proliferation of CD4^+^ T cells [15], [16], and compelling evidence from the experiment of Marsland *et al.* demonstrated that CpG DNA stimulation could activate CD4^+^ T cells from PKC-θ^−/−^ mice and causing EAE, indicating that TLR stimulation could support the activation and differentiation of CD4^+^ T cells in the absence of TCR signaling [17]. TLRs are also involved in the activation and function of nTregs. Direct stimulation of mice CD4^+^ nTregs with TLR2 ligand Pam3Cys increased the proliferation and concomitantly abrogated the function of the cells [18], [19], while stimulation of human nTregs with TLR4 ligand LPS and IL-2 up-regulated FOXP3 expression and the suppressive function [20]. *In vivo* result from TLR9^−/−^ mice also suggested that TLR9 signaling enhanced nTregs function through induction of IDO [21].

TLR5 is expressed in both CD4^+^ T cells and nTregs [22], [23]. Since the TLR5 ligand, flagellin, is commonly expressed in different bacteria species [24], [25], TLR5 may be particularly important to the induction of tolerance to intestinal commensal bacteria and of oral tolerance [26]. Currently, there is only a single report investigated on the direct effect of TLR5-related signals on human nTregs. Crellin *et al.* reported that stimulation of human nTregs with anti-CD3/CD28 and flagellin up-regulated FOXP3 expression and the suppressive function [27]. Since the direct effect of TLR5-related signals on iTregs remains unexamined, the function of TLR5 in human iTregs is investigated in this study.

Previously our laboratory has developed a simple and cost effective novel protocol of large-scale *in vitro* induction and expansion of human alloantigen specific CD4^hi^CD25^+^ regulatory T cells with therapeutic potential from naïve CD4^+^CD25^−^CD45RO^−^ precursors using human allogeneic CD40-activated B cells as stimulators without the use of exogenous cytokine. Co-culture of human naïve CD4^+^CD25^−^ T cells with allogeneic CD40-activated B cells at T cell to B cell ratio of 10∶1 induced a population of CD4^hi^CD25^+^ regulatory T cells [28]. The CD4^hi^CD25^+^ T cells were alloantigen specific CD45RO^+^CCR7^−^CD62L^+^ memory T cells and expressed FOXP3, IFN-γ, CTLA-4, and GITR [28], [29]. Suppressive MLR experiment demonstrated that these cells could suppress T cell proliferation in a cell-cell contact dependent manner which was partially dependent on the surface CTLA-4, indicating that these cells are iTregs [28]. In this experiment, we investigated the role of TLR5-related signals in the generation and function of human CD4^hi^CD25^+^ regulatory T cells induced by allogeneic CD40-activated B cells and have unveiled a novel function of TLR5-related signaling in iTregs. Our results indicated that TLR5-related signaling enhances the proliferation but not the suppressive function of human CD4^hi^CD25^+^ regulatory T cells induced by allogeneic CD40-activated B cells.

## Materials and Methods

### Ethics Statement

Written consent for the use of buffy coat for research purposes was obtained from the donors by the Hong Kong Red Cross Blood Transfusion Services at the time of blood donation. The use of buffy coat for this experiment was approved by the Institutional Review Board of the University of Hong Kong/Hospital Authority Hong Kong West Cluster (IRB Reference Number: UW 07-390).

### Generation of CD40-activated B Cells

PBMC were isolated from buffy coat of healthy adult donors from the Hong Kong Red Cross Blood Transfusion Services. CD40-acitvated B cells were generated from PBMC via CD40 stimulation using lethally irradiated (96Gy) NIH3T3 cells transfected with the human CD40 ligand (t-CD40-L cells) as stimulator in B cell medium as we described previously [30], [31]. Briefly, isolated PBMC were co-cultured with the lethally irradiated (96Gy) t-CD40-L cells in IMDM (GIBCO-BRL, Life Technologies, CA) supplement with the 2ng/ml of IL-4 (R&D systems, MN), 5.5×10^−7^ M of cyclosporine A (Sigma-Aldrich, MO), 50 µg/ml of transferrin (Sigma-Aldrich, MO), 5 µg/ml of insulin (Sigma-Aldrich, MO), 15 µg/ml of gentamycin (GIBCO-BRL, Life Technologies, CA), and 10% of heat-inactivated human AB serum (Innovative Research, MI) at 37°C in 5% CO_2_. Cells were sub-cultured to new 6-well plates of t-CD40-L cells every 3 to 4 days. After 14 days of co-culture, more than 95% of the viable suspended cells are routinely CD19 positive. These B cells were cryopreserved in 10% DMSO medium for future use.

### Isolation of Naïve CD4^+^CD25^−^CD45RO^−^ T Cells and Induction of CD4^hi^CD25^+^ Regulatory T Cells

The CD4^hi^CD25^+^ regulatory T cells were induced by the co-culture of the CD4^+^CD25^−^CD45RO^−^ T cells with the allogeneic CD40-activated B cells at a T-cell: B-cell ratio of 10∶1 for 6 days as described previously [28] unless otherwise specified. Human naïve CD4^+^CD25^−^CD45RO^−^ T cells were isolated from healthy donors PBMC by CD4^+^ T cell enrichment using the human CD4 T Cells Enrichment Cocktails (StemCell Technologies, Canada), followed by negative selection using a human naïve CD4^+^ T Cell Isolation Kit and LD Column (Miltenyi Biotec, Germany) according to manufacturer’s instructions.

### TLR5 Blockade and Chemical Inhibition of ERK1/2 Phosphorylation

10 µg/ml of anti-TLR5 mAb, and its relevant isotype control (Invivogen, CA) were used for the blockade of TLR5. 20 µM of PD98059 and its solvent control DMSO (Merck, Germany) were used for chemical inhibition of ERK1/2 phosphorylation. Antibodies and PD98059 were added to CD4^+^CD25^−^CD45RO^−^ T cells one hour before co-culturing with allogeneic CD40-activated B cells and were replenished when cell culture medium was changed.

### Flow Cytometric Assays

All fluorescence-conjugated antibodies were from BD-Biosciences unless otherwise specified: CD4-pacific blue (Biolegend, CA), CD25-APC-Cy7, CTLA-4-PE, GITR-PE, TLR5-PE (Imgenex, CA), human Foxp3 staining kit (clone: PCH101) (eBiosciences, CA), p-p44/42 (Thr202/Tyr204)-AlexaFluor-488 (Cell Signaling, MA). Annexin V/propidium iodide (Gibco-BRL, Life Technologies, CA) was used for measuring apoptosis. Propidium iodide (Gibco-BRL, Life Technologies, CA) was used for cell cycle analysis. For measuring cell proliferation, naïve CD4^+^CD25^−^CD45RO^−^ T cells were stained with CFSE before co-culturing with allogeneic CD40-activated B cells. Cells were analyzed using FACS LSRII (BD Biosciences, CA) and results were analyzed using FlowJo v8.8.2 (Tree Star, OR). Cell cycle analysis results were analyzed using ModFit (Verity Software House, ME).

### Mixed Lymphocyte Reaction (MLR) Assays

CD4^hi^CD25^+^ regulatory T cells generated with or without the blockade of TLR5 were sorted using FACSAria after 9 days of co-culture. The sorted CD4^hi^CD25^+^ regulatory T cells were titrated and co-cultured with 5×10^4^ responder CD4^+^CD25^−^ T cells from the same donor of the CD4^hi^CD25^+^ regulatory T cells and 5×10^4^ γ-irradiated target PBMC from the donor of the CD40-activated B cells as stimulator for 3 days. ^3^H-thymidine was added to the co-culture in the last 18 hours and the proliferation was analyzed by^ 3^H-thymidine incorporation assay as we described previously [28].

### Statistical Analysis

Graphs and statistical analysis were performed using Prism 5.0 for Windows software (GraphPad Software, CA). One-way ANOVA with Tukey’s pairwise comparisons was used for comparing the percentage of regulatory T cells, apoptotic T cells, and percentage of CD4^hi^CD25^+^ regulatory T cells in S phase. *p* value of <0.05 was considered to be significant.

## Results

### TLR5-related Signals Enhance the Generation of CD4^hi^CD25^+^ Regulatory T Cells Independent of Cell Apoptosis

**Figure 1:**
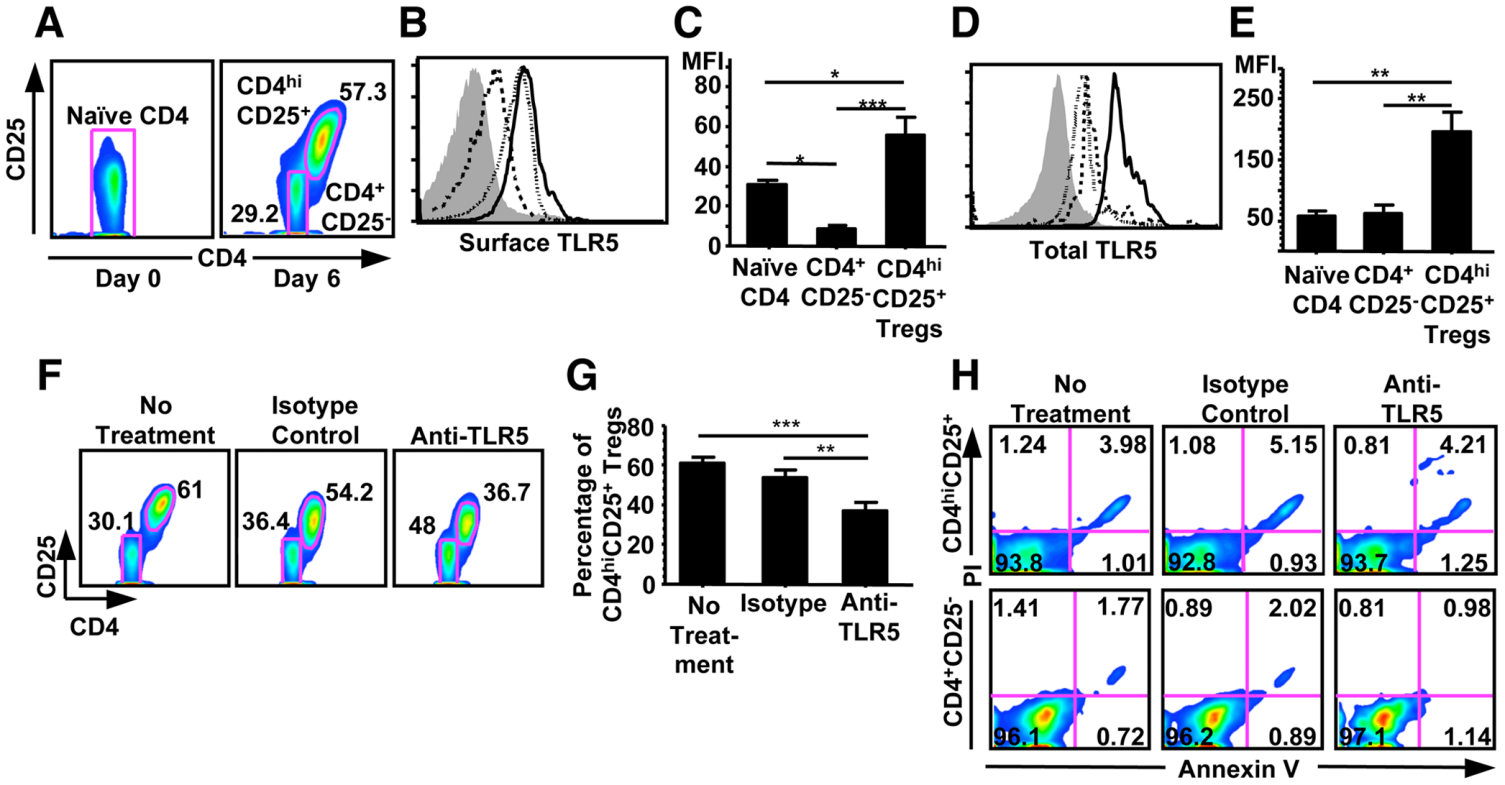
LR5 blockade reduced the generation of CD4^hi^CD25^+^ regulatory T cells and was independent of apoptosis. (A) Flow cytometric analysis of the percentage of CD4^hi^CD25^+^ regulatory T cells generated on Day 6 (right panel) from naïve CD4^+^CD25^−^CD45RO^−^ T cells (left panel). (B) Flow cytometric analysis of the expression of surface TLR5 in freshly isolated naïve CD4^+^CD25^−^CD45RO^−^ T cells (dotted line), and CD4^+^CD25^−^ (dashed line) and CD4^hi^CD25^+^ regulatory T cells (solid line) after 6 days of co-culture of naïve CD4^+^CD25^−^CD45RO^−^ T cells with allogeneic CD40-activated B cells. Filled histogram indicates the staining obtained from isotype-matched mAb controls. (C) Mean fluorescence intensity (MFI) of the expression of surface TLR5. Data show Mean+SEM, n  = 6. (D) Flow cytometric analysis of total TLR5 in freshly isolated naïve CD4^+^CD25^−^CD45RO^−^ T cells (dotted line), CD4^+^CD25^−^ (dashed line), and CD4^hi^CD25^+^ regulatory T cells (solid line) after 6 days of co-culture of naïve CD4^+^CD25^−^CD45RO^−^ T cells with allogeneic CD40-activated B cells. Filled histogram indicates the staining obtained from isotype-matched mAb control. (E) Mean fluorescence intensity (MFI) of the expression of total TLR5. Data show Mean+SEM, n  = 6. (F) Flow cytometric analysis of the generation of CD4^hi^CD25^+^ regulatory T cells with no treatment (left panel), with isotype-matched mAb (middle panel), and with anti-TLR5 blocking mAb (right panel) during the co-culture. (G) Mean percentage of CD4^hi^CD25^+^ regulatory T cells generated with no treatment, with isotype-matched mAb, and with anti-TLR5 blocking mAb. Data shown Mean+SEM, n  = 6. (H) Flow cytometric analysis of the percentage of apoptotic CD4^hi^CD25^+^ regulatory T cells (upper panel) or CD4^+^CD25^−^ T cells (lower panel) after 6 days of co-culture of naïve CD4^+^CD25^−^CD45RO^−^ T cells with allogeneic CD40-activated B cells. All results shown are representative of three independent experiments. *p<0.05, **p<0.01, ***p<0.001, one way ANOVA with Tukey’s pairwise comparisons.

We first investigated the TLR5 expression in the CD4^hi^CD25^+^ regulatory T cells. A population of CD4^hi^CD25^+^ regulatory T cells and a population of CD4^+^CD25^−^ T cells without any regulatory function could be identified in the co-culture of naïve CD4^+^CD25^−^CD45RO^−^ T cells with allogeneic CD40-activated B cells for 6 days (Figure 1A). As shown in Figure 1 B-E, CD4^hi^CD25^+^ regulatory T cells exhibited an up-regulated surface (Figure 1B and C) and total TLR5 protein expression (Figure 1D and E). Interestingly, in CD4^+^CD25^−^ T cells, surface TLR5 expression level was lower than that of naïve CD4^+^CD25^−^CD45RO^−^ T cells while total TLR5 expression was the same (Figure 1 B-E).

The up-regulated TLR5 expression in CD4^hi^CD25^+^ regulatory T cells prompted us to investigate the effect of TLR5-related signals on the generation and function of CD4^hi^CD25^+^ regulatory T cells. It was found that the blockade of TLR5 using anti-TLR5 blocking antibodies decreased CD4^hi^CD25^+^ regulatory T cells generation (Figure 1F and G). Frequency of CD4^hi^CD25^+^ regulatory T cells decreased from 61% of total CD4^+^ T cells to about 36% after 6 days of co-culture (*p*<0.001) (Figure 1G), indicating that TLR5 signaling was involved in CD4^hi^CD25^+^ regulatory T cells generation. Since TLR5 was reported to be anti-apoptotic [32], and could promote the survival of cells and mice subjected to lethal irradiation [33], [34], we further studied whether the reduced CD4^hi^CD25^+^ regulatory T cells generation was due to increased apoptosis of CD4^+^ T cells. Surprisingly, cell death analysis using annexin V/podium iodide staining indicated that the blockade of TLR5 did not increase the apoptosis of either CD4^hi^CD25^+^ regulatory T cells or CD4^+^CD25^−^ T cells. Approximate 5% of CD4^+^CD25^−^ T cells and 2% of CD4^hi^CD25^+^ regulatory T cells were in either early or late apoptotic phase and TLR5 blockade did not alter the percentage (Figure 1H). These results indicated that the reduction of CD4^hi^CD25^+^ regulatory T cell generation by blocking TLR5-related signals is not dependent on cell apoptosis.

### TLR5-related Signals Endorse the Proliferation of CD4^hi^CD25^+^ Regulatory T Cells by Promoting the Process of S Phase

**Figure 2:**
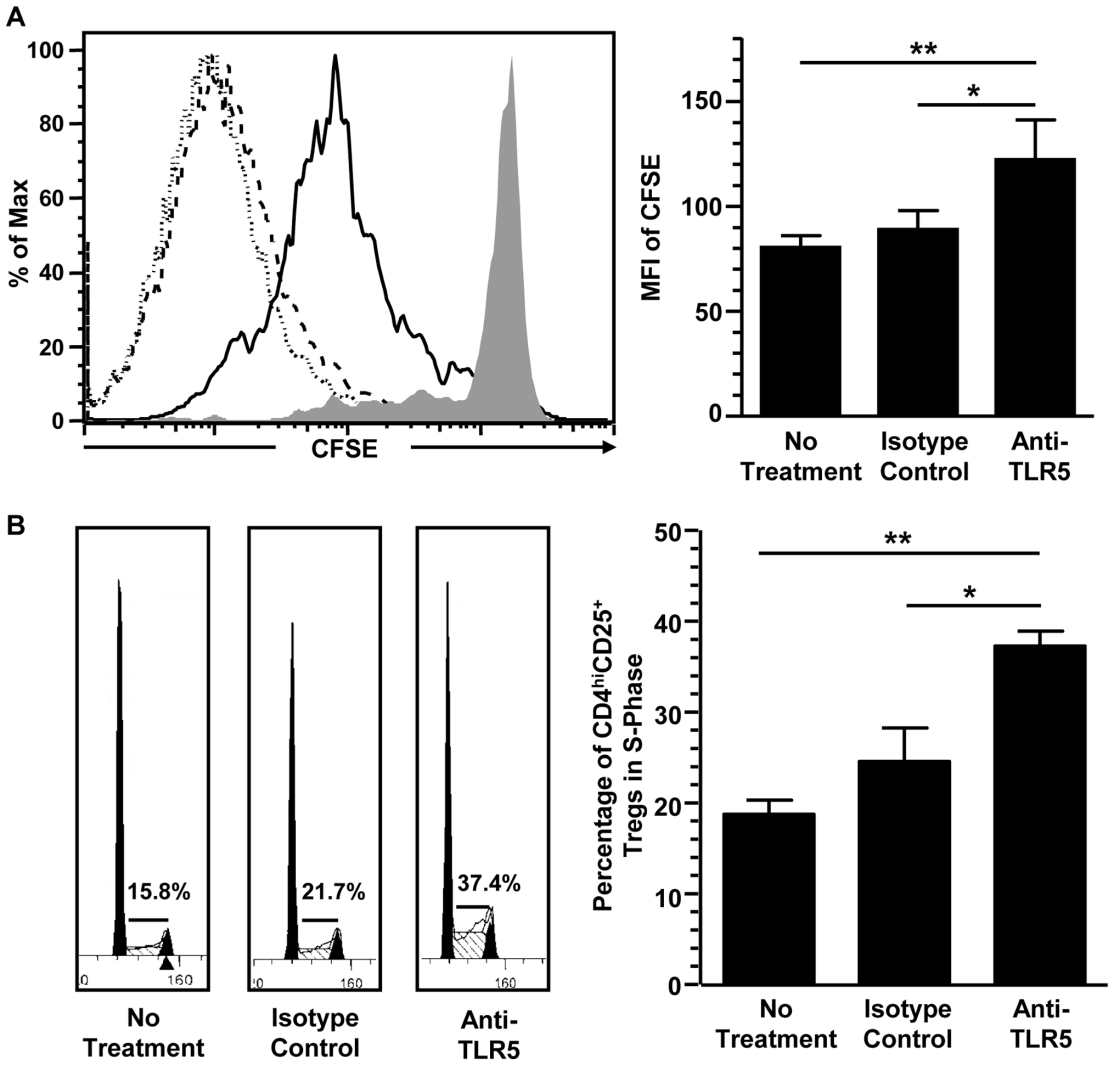
The blockade of TLR5 reduced CD4^hi^CD25^+^ regulatory T cells proliferation by inducing S phase arrest. (A) Flow cytometric analysis of the CFSE signal in CD4^hi^CD25^+^ regulatory T cells generated with no treatment (dotted line), with isotype-matched mAb (dashed line), and with anti-TLR5 blocking mAb (solid line). Filled histogram is the CFSE signal on Day 0 (left panel). Statistical analysis of the MFI of the CFSE in CD4^hi^CD25^+^ regulatory T cells. Data show Mean+SEM, n  = 6. (right panel). (B) Cell cycle analysis of CD4^hi^CD25^+^ regulatory T cells generated with no treatment (left), with isotype-matched mAb (middle), and with anti-TLR5 blocking mAb (right). Numbers indicate the percentage of CD4^hi^CD25^+^ regulatory T cells in S phase (left panel). Statistical analysis of percentage of CD4^hi^CD25^+^ regulatory T cells in S phase. Data show Mean+SEM, n  = 6 (right panel). All data shown are representative from three independent experiments. *p<0.05, **p<0.01, one way ANOVA with Tukey’s pairwise comparisons.

Unaltered apoptosis of CD4^+^ T cells after the blockade of TLR5 suggested that the reduced CD4^hi^CD25^+^ regulatory T cells generation was the result of decreased CD4^+^ T cells proliferation. CFSE staining demonstrated that CD4^hi^CD25^+^ regulatory T cells underwent extensive proliferation and blockade of TLR5 reduced their proliferation (Figure 2A, left panel). The mean fluorescence intensity (MFI) of the CFSE in CD^hi^CD25^+^ regulatory T cells generated without any treatment or with isotype matched mAb were about 80.5 and 89.1 respectively on Day 5. TLR5 blockade increased the MFI to about 122.3, indicating a reduction in proliferation of the CD4^hi^CD25^+^ regulatory T cells (*p*<0.05) (Figure 2A, right panel). This result supported our hypothesis that TLR5 blockade decreased the generation of CD4^hi^CD25^+^ regulatory T cells by reducing its proliferation. Since cell proliferation is a direct result of cell cycle, effect of TLR5 blockade on cell cycle progress of CD4^hi^CD25^+^ regulatory T cells was investigated. After co-culture with allogeneic CD40-activated B cells, about 15% of CD4^hi^CD25^+^ regulatory T cells were in S phase whereas their percentage was increased to about 40% with the blockade of TLR5 (*p*<0.05) (Figure 2B), indicating an arrest in S phase. Therefore, it is concluded that TLR5-related signals enhanced the proliferation of CD4^hi^CD25^+^ regulatory T cells by promoting the process of S phase.

### Reduced ERK1/2 Signaling by the Blockade of TLR5 might Contribute to S Phase Arrest in CD4^hi^CD25^+^ Regulatory T Cells

**Figure 3:**
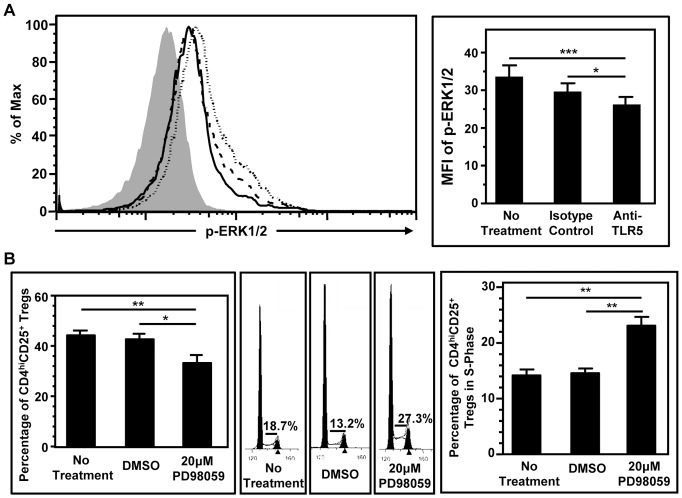
Reduced phosphorylated ERK1/2 might contribute to S phase arrest in CD4^hi^CD25^+^ regulatory T cells. (A) Flow cytometric analysis of the expression of phosphorylated ERK1/2 in CD4^hi^CD25^+^ regulatory T cells generated with no treatment (dotted line), isotype-matched mAb (dashed line), and with anti-TLR5 blocking mAb (solid line). Filled histogram is the staining obtained from isotype-matched mAb control for staining antibody (left panel). Statistical analysis of the MFI of p-ERK1/2 in CD4^hi^CD25^+^ regulatory T cells. Data show Mean+SEM, n  = 10. All data shown are representative from five independent experiments (right panel). (B) Statistical analysis of the percentage of CD4^hi^CD25^+^ regulatory T cells generated on Day 6 with or without the inhibition of ERK1/2 phosphorylation by PD98059. DMSO treated group is the control for PD98059. Data show Mean+SEM, n  = 6. All results shown are from 3 independent experiments (left panel). Cell cycle analysis of CD4^hi^CD25^+^ regulatory T cells generated on Day 6 with or without the inhibition of ERK1/2 phosphorylation by PD98059. DMSO treated group is the control for PD98059. Numbers indicate the percentage of CD4^hi^CD25^+^ regulatory T cells in S phase. All results shown are from 3 independent experiments (middle panel). Mean percentage of CD4^hi^CD25^+^ regulatory T cells in S phase with the inhibition of ERK1/2 phosphorylation by PD98059. Data show Mean+SEM, n  = 6. All results shown are from 3 independent experiments (right panel). *p<0.05, **p<0.01, ***p<0.001, one way ANOVA with Tukey’s pairwise comparisons.

To elucidate the molecular mechanism of the TLR5-blockade induced-S phase arrest, the ERK1/2 phosphorylation was investigated [35]. Flow cytometric analysis indicated that the blockade of TLR5 reduced phosphorylated ERK1/2 (p-ERK1/2) in CD4^hi^CD25^+^ regulatory T cells (Figure 3A, left panel). The MFI of p-ERK1/2 in CD4^hi^CD25^+^ regulatory T cells generated without any treatment or with isotype matched mAb were about 33.6 and 29.7 respectively. TLR5 blockade decreased the MFI to about 26.3 (*p*<0.05) (Figure 3A, right panel), indicating that TLR5 blockade reduced the phosphorylation of ERK1/2. This also suggested that phosphorylation of ERK1/2 might contribute to the S phase arrest. To confirm this, the effect of ERK1/2 phosphorylation inhibition on the generation and the cell cycle progress of CD4^hi^CD25^+^ regulatory T cells were investigated using MEK1/2 inhibitor PD98059 [36]. Naïve CD4^+^CD25^−^CD45RO^−^ T cells were co-cultured with allogeneic CD40-activated B cells in the presence of PD98059. Inhibition of ERK1/2 phosphorylation decreased the generation of CD4^hi^CD25^+^ regulatory T cells from about 45% to about 35% (*p*<0.05) (Figure 3B, left and middle panel), and the percentage of CD4^hi^CD25^+^ regulatory T cells in S phase increased from about 18% to about 28% (Figure 3B, right panel). Taken together, these results indicated that reduced ERK1/2 phosphorylation might contribute to TLR5-blockade induced-S phase arrest.

### TLR5-related Signals do not Affect CD4^hi^CD25^+^ Regulatory T Cells Function

**Figure 4:**
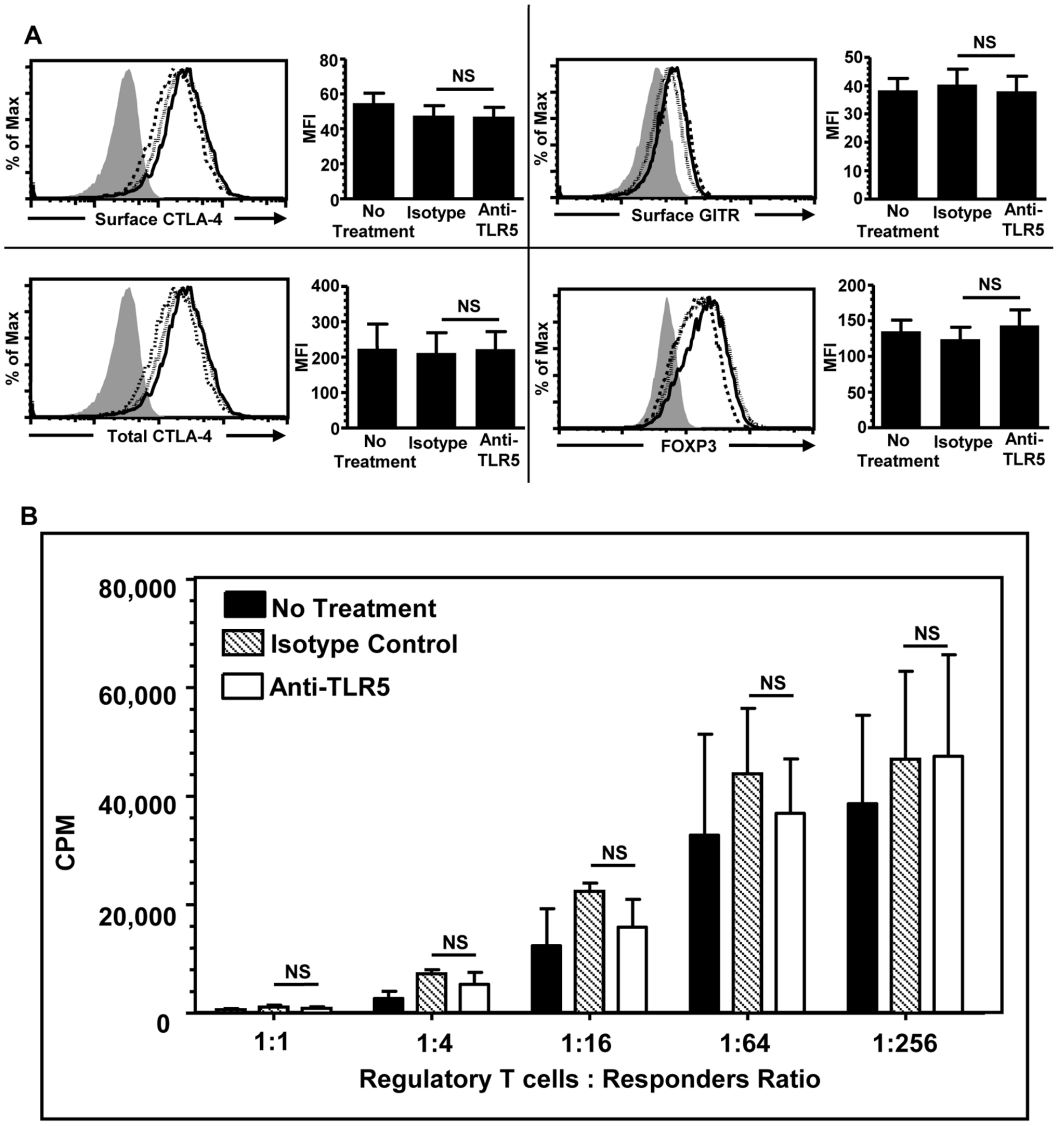
TLR5-related signals did not affect the function of CD4^hi^CD25^+^ regulatory T cells. (A). Flow cytometric and statistical analysis of the expression of surface CTLA-4 (upper left panel), intracellular CTLA-4 (lower left panel), surface GITR (upper right panel), and FOXP3 (lower right panel) of CD4^hi^CD25^+^ regulatory T cells generated with no treatment (dotted line), with isotype-matched mAb (dashed line), and with anti-TLR5 blocking mAb (solid line) after 6 days of co-culture of naïve CD4^+^CD25^−^CD45RO^−^ T cells and allogeneic CD40-activated B cells and filled histogram indicates staining obtained from the isotype-matched mAb for staining antibodies. Data were shown in Mean+SEM, n  = 6. (B) ^3^H-thymidine incorporation of CD4^hi^CD25^+^ regulatory T cells in suppressive MLR at different regulatory T cells: responsders ratio. Data show Mean+SEM, n  = 6. All data shown are from 3 independent experiments. NS, not significant, one way ANOVA with Tukey’s pairwise comparisons.

Previous study by Crellin *et al.* demonstrated that flagellin stimulation up regulated Foxp3 expression and the suppressive function of nTregs [27]. In this study, the effect of TLR5-related signals on the suppressive function of the human allogeneic CD40-activated B cell induced CD4^hi^CD25^+^ regulatory T cells was examined. Previous study from our group showed that the function of CD^hi^CD25^+^ regulatory T cells is partially dependent on the surface expression of CTLA-4 [28]. Therefore, the expression levels of CTLA-4, GITR, and FOXP3 were measured using FACS after TLR5 blockade. It was found that the blockade of TLR5 did not alter the surface and total expression of these molecules and no statistically significant reduction in the MFI of these concerned molecules was detected (Figure 4A). In addition, MLR results indicated that CD4^hi^CD25^+^ regulatory T cells generated in either condition exhibited similar suppressive capacity even at the regulatory T cells: responders ratio of 1∶1 (Figure 4B). Taken together, these data suggested that blockade of TLR5 did not alter the function of CD4^hi^CD25^+^ regulatory T cells.

## Discussion

In this study, we demonstrated that TLR5 signaling was involved in the generation but not the function of human allogeneic CD40-activated B cells induced CD4^hi^CD25^+^ regulatory T cells. Our data provided interesting information about the function of TLR5-related signals in iTregs. This, to the best of our knowledge, is the first report concerning TLR5-related signals in iTregs.

Here we found an increase of TLR5 expression in CD4^hi^CD25^+^ regulatory T cells. This was probably the consequence of CD4^+^ T cell activation during the co-culture. NF-κB and AP-1 binding sites are situated around the promoter region of TLR5 locus [37]. NF-κB and AP-1 are synthesized during T cell activation [38], [39] and may bind to the promoter of TLR5, resulting in the transcription of TLR5. Interestingly, TLR5 can also activate the synthesis of NF-κB and AP-1 [14], thus it is possible that TLR5 was activated during the co-culture and positively feedback to the TLR5 expression. Since TLR5 expression was also up regulated in resting nTregs [22], it is possible that Foxp3 also up regulate the TLR5 expression but the precise mechanism remains to be investigated.

In this study, we further found that blockade of TLR5 using anti-TLR5 blocking antibody reduced the proliferation of CD4^hi^CD25^+^ regulatory T cells through S phase arrest but did not increase the apoptosis of CD4^hi^CD25^+^ regulatory T cells or CD4^+^CD25^−^ T cells. Since TLR5 was reported to be anti-apoptotic [40], it was surprising that blockade of TLR5 did not increase the apoptosis of the cells. This may be explained by the observation from our previous investigation that large amount of IL-2 was produced by the CD40-activated B cells [28], thus it is possible that these IL-2 molecules rescued the CD4^+^ T cells from apoptosis.

The S phase arrest of the CD4^hi^CD25^+^ regulatory T cells may be explained by the associated reduction of the ERK1/2 phosphorylation after TLR5 blockade. It is known that S phase exit or G2/M phase entry is controlled by cdk2 and cyclin A [41], the cdk2 is in turn activated by cdc25a [42], which could be activated and phosphorylated by p-ERK1/2 [43]. Therefore, it is speculated that the reduced ERK1/2 phosphorylation in the CD4^hi^CD25^+^ regulatory T cells decreased the expression and activation of cdc25a, thus in turn, the cdk2 activation, causing S phase arrest. However, the precise molecular mechanism between the reduced ERK1/2 phosphorylation and the S phase arrest remains to be elucidated. In addition, the reduced proliferation of the CD4^hi^CD25^+^ regulatory T cells may also be the result of reduced production of different cytokines. It was reported that stimulation of TLR5 using flagellin resulted in IL-8 production in epithelial cells and gastric cancer cells, increasing the proliferation of these cells [44], [45], and the production of IFN-γ [46]. Therefore, it is possible that TLR5-related signals may enhance the production of IFN-γ, which in turn increases the proliferation of CD4^hi^CD25^+^ regulatory T cells. However, the relative importance between cell cycle control and cytokine production in regulating the proliferation of the CD4^hi^CD25^+^ regulatory T cells remains to be elucidated.

Our results demonstrated that TLR5 is not involved in the suppressive function of CD4^hi^CD25^+^ regulatory T cells. This is in contrast with previous studies by Crellin *et al.* that flagellin stimulation of natural regulatory T cells enhanced the FOXP3 expression and function [27]. Maximal Foxp3 expression in peripheral thymic derived regulatory T cells requires signals from TCR [47], CD28 [48], and IL-2 [49]. IL-2 promotes Foxp3 expression by activating STAT5, which binds to the promoter of Foxp3 locus. In Crellin *et al.* experiment, IL-2 was not used to activate nTregs [27] and the nTregs might express a low level of Foxp3, which could be up regulated by flagellin stimulation. Flagellin stimulation promoted AP-1 activation and the binding of AP-1 to the promoter of Foxp3 locus, thus in turn, the transcription of Foxp3 [50] while IL-2 secreted from the CD40-activated B cells [31] might compensate the effect of TLR5 blockade on the function of CD4^hi^CD25^+^ regulatory T cells in our induction system. The lack of IL-2 in Crellin *et al.* experiment may also explain the contrasting results in CD4^hi^CD25^+^ regulatory T cells proliferation. nTregs is hyporesponsive for proliferation and its proliferation requires the co-existence of CD3, CD28, and IL-2 signaling. It is possible that TLR5 signaling was not potent enough to break this hyporesponsiveness. In contrast, the CD4^hi^CD25^+^ regulatory T cells are induced from naïve CD4^+^CD25^−^CD45RO^−^ T cells, which are not hyporesponsive for proliferation, and the strength of TLR5 signaling may be sufficient in promoting the proliferation of CD4^hi^CD25^+^ regulatory T cells.

In conclusion, our results demonstrated that TLR5-related signals were involved in the proliferation of CD4^hi^CD25^+^ regulatory T cells by promoting the progress of S phase arrest and ERK1/2 signaling may be involved. However, TLR5-related signals did not affect the function of CD4^hi^CD25^+^ regulatory T cells. The role of TLR5-related signaling in CD4^hi^CD25^+^ regulatory T cells is more resemble to CD4^+^ effector T cells than CD4^+^ nTregs as reflected by the contrasting responses in proliferation and suppressive function after the blockade of TLR5. However, whether the same phenomenon can be observed in other types of iTregs such as Tr1 and Th3 remains to be elucidated. Our result also indicated that, unlike TLR2, TLR5-related signaling promoted the proliferation of CD4^hi^CD25^+^ regulatory T cells without diminishing the suppressive function [19]. This suggests that flagellin may be a potential ligand for increasing the number of iTregs and suppressing inflammation in organs such as intestines where induced regulatory T cells are abundant.

## References

[1] Lourenco EV, La Cava A (2011) Natural regulatory T cells in autoimmunity. Autoimmunity 44: 33–42. 21091291 10.3109/08916931003782155 PMC3057884

[2] Bilate AM, Lafaille JJ (2012) Induced CD4+Foxp3+ regulatory T cells in immune tolerance. Annu Rev Immunol 30: 733–758. 22224762 10.1146/annurev-immunol-020711-075043

[3] Le Bras S, Geha RS (2006) IPEX and the role of Foxp3 in the development and function of human Tregs. J Clin Invest 116: 1473–1475. 16741571 10.1172/JCI28880 PMC1464917

[4] Kuhn A, Beissert S, Krammer PH (2008) CD4(+)CD25 (+) regulatory T cells in human lupus erythematosus. Arch Dermatol Res 5: 5. 10.1007/s00403-008-0891-9 18985367

[5] Alpdogan O, van den Brink MR (2012) Immune tolerance and transplantation. Semin Oncol 39: 629–642. 23206840 10.1053/j.seminoncol.2012.10.001 PMC3514882

[6] Corthay A (2009) How do regulatory T cells work? Scand J Immunol 70: 326–336. 19751267 10.1111/j.1365-3083.2009.02308.x PMC2784904

[7] Sansom DM, Walker LS (2006) The role of CD28 and cytotoxic T-lymphocyte antigen-4 (CTLA-4) in regulatory T-cell biology. Immunol Rev 212: 131–148. 16903911 10.1111/j.0105-2896.2006.00419.x

[8] Gogishvili T, Luhder F, Goebbels S, Beer-Hammer S, Pfeffer K, et al (2013) Cell-intrinsic and -extrinsic control of Treg-cell homeostasis and function revealed by induced CD28 deletion. Eur J Immunol 43: 188–193. 23065717 10.1002/eji.201242824

[9] Redmond WL, Ruby CE, Weinberg AD (2009) The role of OX40-mediated co-stimulation in T-cell activation and survival. Crit Rev Immunol 29: 187–201. 19538134 10.1615/critrevimmunol.v29.i3.10 PMC3180959

[10] Wang L, Pino-Lagos K, de Vries VC, Guleria I, Sayegh MH, et al (2008) Programmed death 1 ligand signaling regulates the generation of adaptive Foxp3+CD4+ regulatory T cells. Proc Natl Acad Sci U S A 105: 9331–9336. 18599457 10.1073/pnas.0710441105 PMC2442817

[11] Wakamatsu E, Mathis D, Benoist C (2013) Convergent and divergent effects of costimulatory molecules in conventional and regulatory CD4+ T cells. Proc Natl Acad Sci U S A 110: 1023–1028. 23277554 10.1073/pnas.1220688110 PMC3549117

[12] Szymczak-Workman AL, Workman CJ, Vignali DA (2009) Cutting edge: regulatory T cells do not require stimulation through their TCR to suppress. J Immunol 182: 5188–5192. 19380762 10.4049/jimmunol.0803123 PMC2745156

[13] Schmidt-Weber CB, Alexander SI, Henault LE, James L, Lichtman AH (1999) IL-4 enhances IL-10 gene expression in murine Th2 cells in the absence of TCR engagement. J Immunol 162: 238–244. 9886391

[14] Akira S, Uematsu S, Takeuchi O (2006) Pathogen recognition and innate immunity. Cell 124: 783–801. 16497588 10.1016/j.cell.2006.02.015

[15] Gelman AE, Zhang J, Choi Y, Turka LA (2004) Toll-like receptor ligands directly promote activated CD4+ T cell survival. J Immunol 172: 6065–6073. 15128790 10.4049/jimmunol.172.10.6065 PMC2833313

[16] Caron G, Duluc D, Fremaux I, Jeannin P, David C, et al (2005) Direct stimulation of human T cells via TLR5 and TLR7/8: flagellin and R-848 up-regulate proliferation and IFN-gamma production by memory CD4+ T cells. J Immunol 175: 1551–1557. 16034093 10.4049/jimmunol.175.3.1551

[17] Marsland BJ, Nembrini C, Grun K, Reissmann R, Kurrer M, et al (2007) TLR ligands act directly upon T cells to restore proliferation in the absence of protein kinase C-theta signaling and promote autoimmune myocarditis. J Immunol 178: 3466–3473. 17339441 10.4049/jimmunol.178.6.3466

[18] Sutmuller RP, den Brok MH, Kramer M, Bennink EJ, Toonen LW, et al (2006) Toll-like receptor 2 controls expansion and function of regulatory T cells. J Clin Invest 116: 485–494. 16424940 10.1172/JCI25439 PMC1332026

[19] Oberg HH, Ly TT, Ussat S, Meyer T, Kabelitz D, et al (2010) Differential but direct abolishment of human regulatory T cell suppressive capacity by various TLR2 ligands. J Immunol 184: 4733–4740. 20363971 10.4049/jimmunol.0804279

[20] Milkova L, Voelcker V, Forstreuter I, Sack U, Anderegg U, et al (2009) The NF-kappaB signalling pathway is involved in the LPS/IL-2-induced upregulation of FoxP3 expression in human CD4CD25 regulatory T cells. Exp Dermatol 21: 21. 10.1111/j.1600-0625.2009.00953.x 19845758

[21] Fallarino F, Volpi C, Zelante T, Vacca C, Calvitti M, et al (2009) IDO mediates TLR9-driven protection from experimental autoimmune diabetes. J Immunol 183: 6303–6312. 19841163 10.4049/jimmunol.0901577

[22] Kabelitz D (2007) Expression and function of Toll-like receptors in T lymphocytes. Curr Opin Immunol 19: 39–45. 17129718 10.1016/j.coi.2006.11.007

[23] Mansson A, Adner M, Cardell LO (2006) Toll-like receptors in cellular subsets of human tonsil T cells: altered expression during recurrent tonsillitis. Respir Res 7: 36. 16504163 10.1186/1465-9921-7-36 PMC1481585

[24] Fournier B, Williams IR, Gewirtz AT, Neish AS (2009) Toll-like receptor 5-dependent regulation of inflammation in systemic Salmonella enterica Serovar typhimurium infection. Infect Immun 77: 4121–4129. 19596770 10.1128/IAI.00656-09 PMC2738035

[25] Kinnebrew MA, Ubeda C, Zenewicz LA, Smith N, Flavell RA, et al (2010) Bacterial flagellin stimulates Toll-like receptor 5-dependent defense against vancomycin-resistant Enterococcus infection. J Infect Dis 201: 534–543. 20064069 10.1086/650203 PMC2811237

[26] Sun J, Fegan PE, Desai AS, Madara JL, Hobert ME (2007) Flagellin-induced tolerance of the Toll-like receptor 5 signaling pathway in polarized intestinal epithelial cells. Am J Physiol Gastrointest Liver Physiol 292: G767–778. 17138965 10.1152/ajpgi.00447.2006

[27] Crellin NK, Garcia RV, Hadisfar O, Allan SE, Steiner TS, et al (2005) Human CD4+ T cells express TLR5 and its ligand flagellin enhances the suppressive capacity and expression of FOXP3 in CD4+CD25+ T regulatory cells. J Immunol 175: 8051–8059. 16339542 10.4049/jimmunol.175.12.8051

[28] Tu W, Lau YL, Zheng J, Liu Y, Chan PL, et al (2008) Efficient generation of human alloantigen-specific CD4+ regulatory T cells from naive precursors by CD40-activated B cells. Blood 112: 2554–2562. 18599794 10.1182/blood-2008-04-152041 PMC2532818

[29] Zheng J, Liu Y, Qin G, Lam KT, Guan J, et al (2011) Generation of human Th1-like regulatory CD4(+) T cells by an intrinsic IFN-gamma- and T-bet-dependent pathway. Eur J Immunol 41: 128–139. 21182084 10.1002/eji.201040724

[30] Zheng J, Liu Y, Liu M, Xiang Z, Lam KT, et al (2013) Human CD8+ regulatory T cells inhibit GVHD and preserve general immunity in humanized mice. Science translational medicine 5: 168ra169. 10.1126/scitranslmed.3004943 23325802

[31] Zheng J, Liu Y, Lau YL, Tu W (2010) CD40-activated B cells are more potent than immature dendritic cells to induce and expand CD4(+) regulatory T cells. Cellular & molecular immunology 7: 44–50. 20081875 10.1038/cmi.2009.103 PMC4003254

[32] Salamone GV, Petracca Y, Fuxman Bass JI, Rumbo M, Nahmod KA, et al (2010) Flagellin delays spontaneous human neutrophil apoptosis. Lab Invest 90: 1049–1059. 20368700 10.1038/labinvest.2010.77

[33] Neish AS (2007) TLRS in the gut. II. Flagellin-induced inflammation and antiapoptosis. Am J Physiol Gastrointest Liver Physiol 292: G462–466. 17082224 10.1152/ajpgi.00274.2006

[34] Burdelya LG, Krivokrysenko VI, Tallant TC, Strom E, Gleiberman AS, et al (2008) An agonist of toll-like receptor 5 has radioprotective activity in mouse and primate models. Science 320: 226–230. 18403709 10.1126/science.1154986 PMC4322935

[35] Huang F, Xiong X, Wang H, You S, Zeng H (2010) Leptin-induced vascular smooth muscle cell proliferation via regulating cell cycle, activating ERK1/2 and NF-kappaB. Acta biochimica et biophysica Sinica 42: 325–331. 20458445 10.1093/abbs/gmq025

[36] Nishimoto S, Nishida E (2006) MAPK signalling: ERK5 versus ERK1/2. EMBO reports 7: 782–786. 16880823 10.1038/sj.embor.7400755 PMC1525153

[37] (2000) Champion ChiP Transcription Factor Search Portal. 2012–05–12 ed. CA: Qiagen.

[38] Lupino E, Ramondetti C, Piccinini M (2012) IkappaB kinase beta is required for activation of NF-kappaB and AP-1 in CD3/CD28-stimulated primary CD4(+) T cells. Journal of immunology 188: 2545–2555. 10.4049/jimmunol.1102938 22331067

[39] Schmidt A, Oberle N, Weiss EM, Vobis D, Frischbutter S, et al (2011) Human regulatory T cells rapidly suppress T cell receptor-induced Ca(2+), NF-kappaB, and NFAT signaling in conventional T cells. Sci Signal 4: ra90. 22375050 10.1126/scisignal.2002179

[40] Vijay-Kumar M, Wu H, Jones R, Grant G, Babbin B, et al (2006) Flagellin suppresses epithelial apoptosis and limits disease during enteric infection. Am J Pathol 169: 1686–1700. 17071592 10.2353/ajpath.2006.060345 PMC1780197

[41] Schafer KA (1998) The cell cycle: a review. Vet Pathol 35: 461–478. 9823588 10.1177/030098589803500601

[42] Collins K, Jacks T, Pavletich NP (1997) The cell cycle and cancer. Proc Natl Acad Sci U S A 94: 2776–2778. 9096291 10.1073/pnas.94.7.2776 PMC34145

[43] Chen S, Gardner DG (2004) Suppression of WEE1 and stimulation of CDC25A correlates with endothelin-dependent proliferation of rat aortic smooth muscle cells. J Biol Chem 279: 13755–13763. 14742443 10.1074/jbc.M310064200

[44] Steiner TS, Ivison SM, Yao Y, Kifayet A (2010) Protein kinase D1 and D2 are involved in chemokine release induced by toll-like receptors 2, 4, and 5. Cell Immunol 264: 135–142. 20557879 10.1016/j.cellimm.2010.05.012

[45] Song EJ, Kang MJ, Kim YS, Kim SM, Lee SE, et al (2011) Flagellin promotes the proliferation of gastric cancer cells via the Toll-like receptor 5. Int J Mol Med 28: 115–119. 21455558 10.3892/ijmm.2011.656

[46] Simone R, Floriani A, Saverino D (2009) Stimulation of Human CD4 T Lymphocytes via TLR3, TLR5 and TLR7/8 Up-Regulates Expression of Costimulatory and Modulates Proliferation. Open Microbiol J 3: 1–8. 19294011 10.2174/1874285800903010001 PMC2656776

[47] Josefowicz SZ, Wilson CB, Rudensky AY (2009) Cutting edge: TCR stimulation is sufficient for induction of Foxp3 expression in the absence of DNA methyltransferase 1. J Immunol 182: 6648–6652. 19454658 10.4049/jimmunol.0803320

[48] Soligo M, Camperio C, Caristi S, Scotta C, Del Porto P, et al (2011) CD28 costimulation regulates FOXP3 in a RelA/NF-kappaB-dependent mechanism. Eur J Immunol 41: 503–513. 21268019 10.1002/eji.201040712

[49] Haiqi H, Yong Z, Yi L (2011) Transcriptional regulation of Foxp3 in regulatory T cells. Immunobiology 216: 678–685. 21122941 10.1016/j.imbio.2010.11.002

[50] von Boehmer H, Nolting J (2008) What turns on Foxp3? Nat Immunol 9: 121–122. 18204423 10.1038/ni0208-121

